# Genetic Characterization of a Novel HIV-1 Circulating Recombinant Form (CRF74_01B) Identified among Intravenous Drug Users in Malaysia: Recombination History and Phylogenetic Linkage with Previously Defined Recombinant Lineages

**DOI:** 10.1371/journal.pone.0133883

**Published:** 2015-07-21

**Authors:** Hui Ting Cheong, Wei Zhen Chow, Yutaka Takebe, Jack Bee Chook, Kok Gan Chan, Haider Abdulrazzaq Abed Al-Darraji, Clayton Koh, Adeeba Kamarulzaman, Kok Keng Tee

**Affiliations:** 1 Centre of Excellence for Research in AIDS (CERiA), Department of Medicine, Faculty of Medicine, University of Malaya, Kuala Lumpur, Malaysia; 2 AIDS Research Center, National Institute of Infectious Diseases, Toyama, Shinjuku-ku, Tokyo, Japan; 3 School of Medicine, Yokohama City University, Yokohama, Kanagawa, Japan; 4 Division of Genetics and Molecular Biology, Institute of Biological Sciences, Faculty of Science, University of Malaya, Kuala Lumpur, Malaysia; University of Athens, Medical School, GREECE

## Abstract

In many parts of Southeast Asia, the HIV-1 epidemic has been driven by the sharing of needles and equipment among intravenous drug users (IDUs). Over the last few decades, many studies have proven time and again that the diversity of HIV-1 epidemics can often be linked to the route of infection transmission. That said, the diversity and complexity of HIV-1 molecular epidemics in the region have been increasing at an alarming rate, due in part to the high tendency of the viral RNA to recombine. This scenario was exemplified by the discovery of numerous circulating recombinant forms (CRFs), especially in Thailand and Malaysia. In this study, we characterized a novel CRF designated CRF74_01B, which was identified in six epidemiologically unlinked IDUs in Kuala Lumpur, Malaysia. The near-full length genomes were composed of CRF01_AE and subtype B', with eight breakpoints dispersed in the *gag-pol* and *nef* regions. Remarkably, this CRF shared four and two recombination hotspots with the previously described CRF33_01B and the less prevalent CRF53_01B, respectively. Genealogy-based Bayesian phylogenetic analysis of CRF74_01B genomic regions showed that it is closely related to both CRF33_01B and CRF53_01B. This observation suggests that CRF74_01B was probably a direct descendent from specific lineages of CRF33_01B, CRF53_01B and subtype Bʹ that could have emerged in the mid-1990s. Additionally, it illustrated the active recombination processes between prevalent HIV-1 subtypes and recombinants in Malaysia. In summary, we report a novel HIV-1 genotype designated CRF74_01B among IDUs in Kuala Lumpur, Malaysia. The characterization of the novel CRF74_01B is of considerable significance towards the understanding of the genetic diversity and population dynamics of HIV-1 circulating in the region.

## Introduction

Group M, the pandemic branch of HIV-1 can be classified into different subtypes named A-D, F-H, J and K. Recombinant lineages generated through recombination between subtypes isolated from at least three epidemiologically unlinked individuals are known as circulating recombinant forms (CRFs), else these recombinants would be known as unique recombinant forms (URFs) [1].

As of April 2015, at least 72 CRFs have been identified globally (http://www.hiv.lanl.gov/). Although HIV-1 subtypes A, B and C remained the most prevalent genotypes worldwide, CRFs can be attributed to at least 16% of HIV-1 infections globally and are anticipated to increase in proportion [2]. Similar patterns can be observed in Asia, where the complexity and diversity of HIV-1 increases in parallel to the cumulated number of HIV-1 infections, though the rates and extent varies [3, 4].

In Malaysia, the early phase of the HIV-1 epidemic was driven mostly by intravenous drug users (IDUs) that were associated with subtype Bʹ of Thai origin and CRF01_AE. Since then, numerous HIV-1 CRFs such as CRF33_01B, CRF48_01B, CRF52_01B, CRF53_01B, CRF54_01B, and CRF58_01B have been reported in Malaysia in the past decade [5–10], all of which have arisen from the predominant subtype B' and CRF01_AE co-circulating in the country. These new CRFs could in fact further recombine with other subtypes or CRFs, generating second-generation recombinant descendants. One example is the CRF48_01B discovered in 2007, a CRF descended from the CRF33_01B lineage [6]. More recently, a new CRF candidate closely related to CRF33_01B was also detected through molecular screening [11], indicating an increasing trend of second-generation CRFs emerging in the region. Therefore, one could anticipate that HIV-1 infections caused by CRFs would increase not only in proportion, but also in complexity over time [2]. In this study, we described a novel CRF candidate discovered among IDUs in Malaysia.

## Materials and Methods

### Ethics Statement

The study was approved by the University Malaya Medical Centre (UMMC) Medical Ethics Committee. Standard, multilingual consent forms allowed by the Medical Ethics Committee were used. Written consent was obtained from all study participants. Being an especially vulnerable population, all interviews and data collected from study participants were kept confidential. Potential participants who declined to take part in the study were not in any way disadvantaged from receiving medical attention, treatment and care.

### Amplification and genetic characterization of a novel second-generation CRF

Patients with history of unsafe injecting drug practices were recruited in Kuala Lumpur, Malaysia between 2010 and 2011. HIV-1 RNA was extracted from plasma samples through magnetic silica-based method implemented in the automated NucliSENS easyMAG platform (BioMerieux, France). Reverse transcription was carried out with random hexamers using SuperScript III RNase H^-^ Reverse Transcriptase (Invitrogen, California, USA) according to manufacturer’s instruction. Through amplification of the protease-reverse transcriptase (PRRT) genes and neighbour-joining analysis described in another study conducted earlier [11], four of 128 (3.1%) PRRT sequences from epidemiologically-unlinked study subjects forming a distinct cluster were identified. In addition, we identified two other isolates from our routine genotyping, which shares a clade with the four sequences identified previously. In order to study the detailed mosaic structures for this monophyletic cluster, near-full length genome amplification were carried out through nested PCR strategies, using sets of primers described in **S1 Table**. PCR products were purified and sequenced through the ABI PRISM 3730XL DNA Analyzer (Applied Biosystems, California, USA). Nucleotide sequences were assembled to produce near-full length genomes of approximately 9 kb in size.

### Phylogenetic and recombination analysis

In order to investigate the phylogenetic relationship of the sequences, reference sequences relevant to HIV-1 epidemics in Southeast Asia, such as CRF01_AE, subtype B of Western origin, subtype Bʹ of Thai origin, CRF07_BC, CRF08_BC, CRF15_01B, CRF33_01B, CRF34_01B, CRF48_01B, CRF51_01B, CRF53_01B, CRF54_01B and CRF58_01B were downloaded from the Los Alamos National Laboratory HIV sequence database (http://www.hiv.lanl.gov) for phylogenetic analysis. References and query sequences (including near-full length and partial genomes) were codon-aligned in accordance to HIV Sequence Compendium 2013 (http://www.hiv.lanl.gov). Subsequently, bootscanning and informative sites estimation [12] using SimPlot version 3.5.1 [13] were carried out. HIV-1 CRF01_AE (90THCM240) and subtype Bʹ (93CNRL42) were used as putative parental genotypes, as well as subtype C (95IN21068) as an outlier group, to determine the recombination structures as well as the location of breakpoints in the near-full length and partial sequences. Genomic sequences with similar mosaic structures and recombination breakpoints were categorised as novel CRFs. On top of that, breakpoints for the previously reported CRF33_01B and CRF53_01B were also determined concurrently to ascertain the accuracy of the estimated breakpoints for the novel CRF. Next, sub-genomic region neighbor-joining trees were constructed using MEGA version 6, on Kimura two-parameter model with a transition-to-transversion ratio of 2.0 [14] to reaffirm the parental lineages of each sub-genomic segment. The reliability of the branching orders were analysed by bootstrap replicates of 1000. Maximum likelihood tree analyses were performed using PAUP version 4.0 [15].

### Bayesian coalescent analysis

In order to study the phylogenetic relationship between the novel CRF as well as the approximate time of emergence of the novel CRF and other published CRFs in Malaysia, Bayesian coalescent analysis using the Markov chain Monte Carlo (MCMC) sampling method were performed through BEAST version 1.7.5 [16] under the uncorrelated log-normal relaxed clock model with the general time-reversible (GTR) nucleotide substitution model with constant and exponential coalescence model. Each MCMC analysis was executed for 50 million states, sampled at every 50,000 states. The model with effective sampling size (ESS) >200 was selected. Posterior probabilities densities were determined in Tracer v1.5 (available in http://tree.bio.ed.ac.uk/software/tracer/) and 10% of each chain will be discarded as burn-in. All sequences studied have been deposited in GenBank, as listed in **Table 1**.

**Table 1:** Demographic characteristics of study subjects infected by HIV-1 CRF74_01B.

Sample ID	Gender	Risk group	Collection year	HXB2 location (nt)	Accession number
10MYKJ052	Male	IDU*	2010	643–9606	KR019770
10MYPR268	Male	IDU	2010	695–9606	KR019771
11MYPR416	Male	IDU	2011	682–9533	KR019772
10MYKJ016	Male	IDU	2010	733–4898^#^	KR019773
11MYJJ741	Male	IDU	2011	708–4898^#^	KR019774
11MYYC672	Male	IDU	2011	708–4898^#^	KR019775

* IDU, intravenous drug users

^#^ Partial genome

## Results and Discussion

### Characterization of novel HIV-1 CRF74_01B isolated in Kuala Lumpur, Malaysia

**Fig 1:**
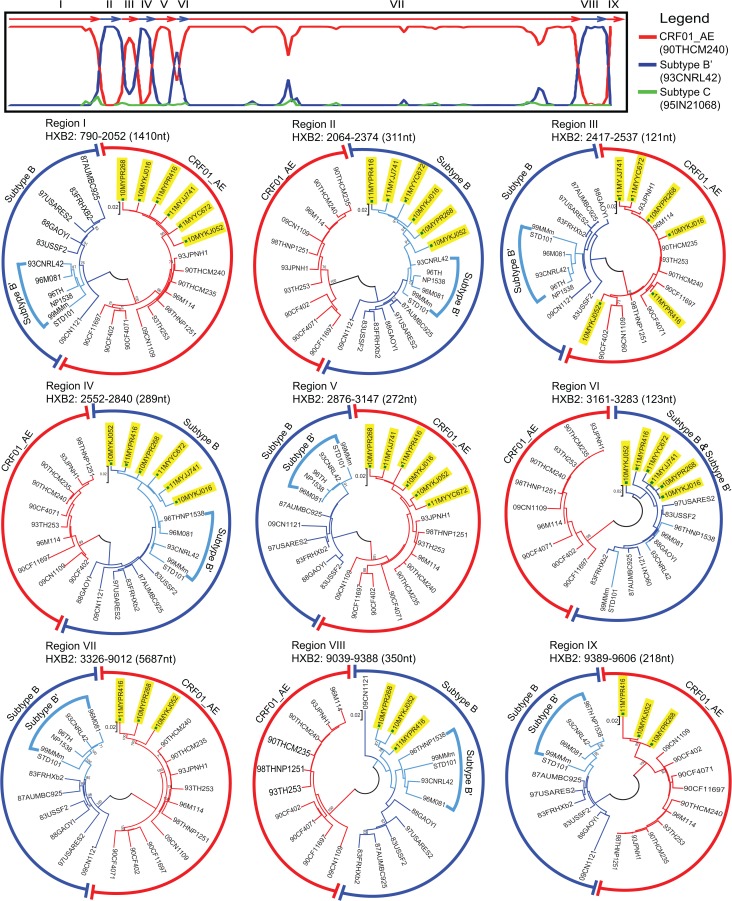
Recombination and neighbour joining analysis of HIV-1 CRF74_01B. The mosaic structure of CRF74_01B was depicted through bootscan using HIV-1 CRF01_AE (90THCM240) and subtype Bʹ (93CNRL42) as putative parental subtypes, and subtype C (95IN21068) as outlier strain. Subtype assignment for sub-genomic regions of CRF74_01B (regions I to IX) were determined and depicted using neighbour joining analysis. Red branches indicate CRF01_AE while blue branches indicate subtype B and Bʹ reference strains. CRF74_01B isolates were indicated (yellow shades).

**Fig 2:**
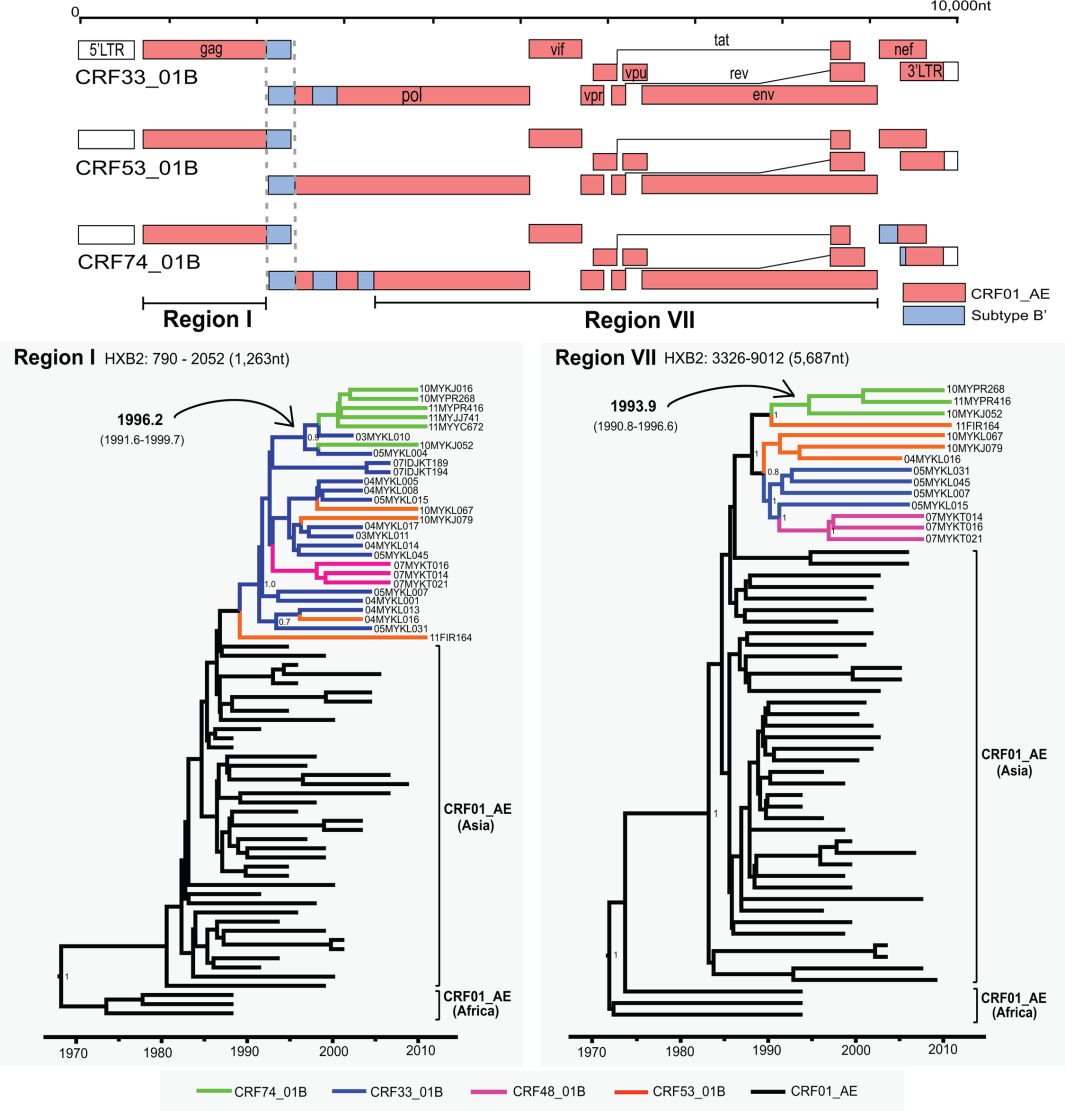
Recombination structures and sub-genomic maximum clade credibility tree reconstructions. Recombination structures of HIV-1 CRF33_01B, CRF53_01B and CRF74_01B were shown. Shared recombination breakpoints between these CRFs were indicated by dashed lines. Maximum clade credibility trees for the *gag* region (region I; HXB2: 790–2052) and *pol-env* region (region VII; HXB2: 3326–9012) of CRF74_01B and other relevant reference sequences were reconstructed. The means of tMRCA of CRF74_01B for region I and region VII were estimate around 1996.2 and 1993.9, respectively, with the 95% credibility intervals stated in parentheses.

A total of six isolates from epidemiologically-unlinked individuals formed a monophyletic cluster based on phylogenetic analysis of the PRRT sequences reported previously [11]. Among these six isolates, three near-full length and three partial genomes were successfully amplified and sequenced (**Table 1**). The near-full length genomes of isolates 10MYKJ052, 10MYPR268 and 11MYPR416 spanned the *gag*, *pol*, *env*, *tat*, *rev*, *vif*, *vpr*, *vpu* and *nef* genes. The partial genomes of isolates 10MYKJ016, 11MYYC672, 11MYJJ741 spanned the *gag* and *pol* region (HXB2: 790–4898) (attempts to amplify the near-full length genomes was unsuccessful due to limited specimens quantity and poor specimens quality). The recombination structures were determined through bootscanning and informative sites analysis. All three near-full length genomes have identical mosaic recombination structures, which are composed of nine sub-regions alternating between CRF01_AE and subtype Bʹ. Sub-region neighbour joining analyses also confirmed the putative parental subtype of each sub-region (**Fig 1**). A total of eight breakpoints were estimated at HXB2 positions 2053–2063, 2375–2416, 2538–2551, 2841–2875, 3148–3160, 3284–3325, 9013–9038 and 9388–9389. Specifically, five sub-regions of CRF01_AE were identified by informative site analysis at HXB2 positions 643 to 2052, 2417 to 2537, 2876 to 3147, 3326 to 9012 and 9389 to 9606, while four sub-regions of subtype Bʹ were observed in 2064 to 2374, 2552 to 2840, 3161 to 3283 and 9039 to 9388. However, we note that sub-region VI, which was only 123bp in size (HXB2 position: 3161 to 3283), did not provide sufficient phylogenetic signal for definitive distinction of subtype B (Western origin) and subtype Bʹ (Thai origin).In particular, the first four recombination breakpoints were similar to those in CRF33_01B, a CRFs previously identified in Malaysia. Similarly, the first two breakpoints were similar to CRF53_01B [8] (**Fig 2**). Recombination and sub-region tree analysis of the three partial genomes (isolates 10MYKJ016, 11MYYC672, 11MYJJ741) also showed identical mosaic structures and shared six recombination breakpoints in the *gag*-*pol* region (HXB2: 790–4898) with the near-full length genomes (10MYKJ052, 10MYPR268 and 11MYPR416) (**Fig 1**). Recombinant structures of these strains are different from any known CRFs reported to date. Taken together, the novel recombination lineage identified in this report was designated CRF74_01B by the HIV Los Alamos National Laboratory according to the standardized nomenclature proposal [1].

### Phylogenetic analysis of CRF74_01B

As sharing of breakpoints typically suggest a common ancestry between recombinants [3, 17], it is possible that CRF74_01B could be a descendent of CRF33_01B and CRF53_01B, or vice versa. The former hypothesis is highly probable, as CRF33_01B has gradually dispersed and established among the general population since its first report in 2006 [11], therefore having a higher chance to co-circulate with other genotypes and subsequently recombine to generate novel CRF. In order to investigate the phylogenetic relationship of CRF74_01B with other structurally related recombinant lineages, the longest shared genetic regions (non-recombinant genetic fragment) with sufficient phylogenetic signal were identified among CRF74_01B, CRF33_01B and CRF53_01B. Regions corresponding to HXB2 positions 790–2052 (similar to region I in **Fig 1**) and 3326–9012 (region VII in **Fig 1**) were selected for maximum clade credibility (MCC) tree reconstructions in BEAST. MCC tree reconstructions under the constant and exponential demographic models produced similar tree topologies, thus only the constant tree model is shown (**Fig 2**).

**Fig 3:**
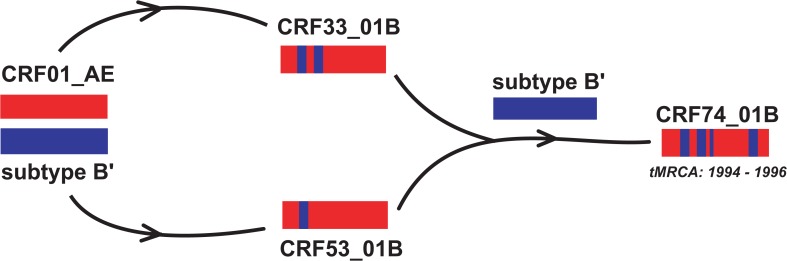
Plausible genetic recombination events leading to the generation of HIV-1 CRF74_01B. Using information on the recombination structures and phylogenetic inference of CRF74_01B and other genetically related CRFs as possible parental lineages, a chronology of recombination events leading to the emergence of CRF74_01B was proposed. The diagram highlights CRF74_01B as a second-generation recombinant with an estimated tMRCA between 1994 and 1996.

The topologies of the MCC tree showed that CRF74_01B branched from an internal node along with CRF33_01B in region I (**Fig 2**), which could indicate a common ancestry between these two CRFs; though one particular strain of CRF74_01B, 10MYKL052, is paraphyletic in relation to other CRF74_01B strains. On the other hand, CRF74_01B appears to form a monophyletic cluster with CRF53_01B in the longer sub-genomic region VII (**Fig 2**). Therefore, it is likely that CRF74_01B is a second-generation progeny descended from specific lineages of CRF33_01B and CRF53_01B. Notably, CRF33_01B and CRF53_01B appear to have emerged from a common ancestor, as both group branches from one common internal node (**Fig 2**). On the other hand, CRF48_01B that was reported to be descended from CRF33_01B [6], showed no direct relationship to CRF74_01B and is clustered independently within CRF33_01B. Similar observations can be derived from maximum likelihood analyses (**S1 Fig**). Based on the Bayesian MCMC analysis in this study, the evolutionary rates for region I and region VII were estimated at 2.3 x 10^−3^ (95% highest posterior density (HPD): 1.8 x 10^−3^–2.8 x 10^−3^) and 2.8 x 10^−3^ (95% HPD: 2.4 x 10^−3^–3.2 x 10^−3^), substitutions per site per year, respectively, which are in concordance with substitution rates estimated elsewhere [18, 19]. Following the MCC trees depicted in **Fig 2**, the estimated time for the most recent common ancestor (tMRCA) for CRF74_01B placed its emergence at approximately between 1994 and 1996. This outcome supports that notion that CRF74_01B emerged a few years after the emergence of its putative parental strain CRF33_01B (**Fig 3**), which is proposed to have emerged between 1991 and 1993 [18].

However, certain limitations that may affect the tMRCA estimation and interpretation in the study should be addressed. For one, the genetic data used for the analyses were limited, as few near-full length genomes of CRF53_01B and CRF74_01B are available due to their low prevalence in the country [20]. Even for an established recombinant such as CRF33_01B, which has an increasingly extensive transmission network in Malaysia [5, 11], only a few near-full length genomes are readily available for comprehensive phylogenetic analyses. Secondly, monophyletic rules [21] suggested that the discrepancies between the two MCC tree topologies could be a result of multiple recombination processes, which could complicate phylogenetic analysis due to limitations in the current theoretical and computational settings [22]. Therefore, until more isolates are detected and sequenced, the intricate relationship between these CRFs must be interpreted with caution, although phylogenetic evidence suggests the ancestral roles of both CRF33_01B and CRF53_01B (**Figs 2**and **3**). In short, CRF74_01B is probably a second-generation recombinant lineage generated via yet undefined recombination event(s) that involved CRF33_01B, CRF53_01B and subtype Bʹ.

The emergence of CRF74_01B further highlights the continuous inter-genotypic recombination processes between the various genotypes and recombinants that are circulating among the various risk groups in Malaysia. It also prompted the necessity of vigilant surveillance on the genetic diversity of HIV-1 epidemics in Malaysia, since it has increased tremendously with the description of the CRF33_01B, CRF48_01B, CRF52_01B, CRF53_01B, CRF54_01B, CRF58_01B and the latest addition of CRF74_01B.

## Conclusion

We identified a novel CRF74_01B among IDUs in Malaysia. Thus far, this is the sixth CRF found in the country. Bayesian MCMC analysis showed that CRF74_01B could be a second-generation progeny lineage from the recombination between CRF33_01B, CRF53_01B and subtype Bʹ. Hence, continuous molecular screening and epidemic surveillance should be implemented in order to comprehend the evolutionary dynamics of the HIV-1 epidemics, which could be crucial in the design of effective vaccines and intervention measures.

## Supporting Information

S1 Fig Maximum likelihood analyses for sub-genomic region I and region VII. (PDF)

S1 Table List of HIV-1 primers for near-full length genome amplification. (DOCX)
