# Supplementary figures and images for: Genetic Characterization of a Novel HIV-1 Circulating Recombinant Form (CRF74_01B) Identified among Intravenous Drug Users in Malaysia: Recombination History and Phylogenetic Linkage with Previously Defined Recombinant Lineages
Source: PLoS One. 2015 Jul 21;10(7):e0133883. doi: 10.1371/journal.pone.0133883 (PMC4510129; doi:10.1371/journal.pone.0133883)

Figure S1

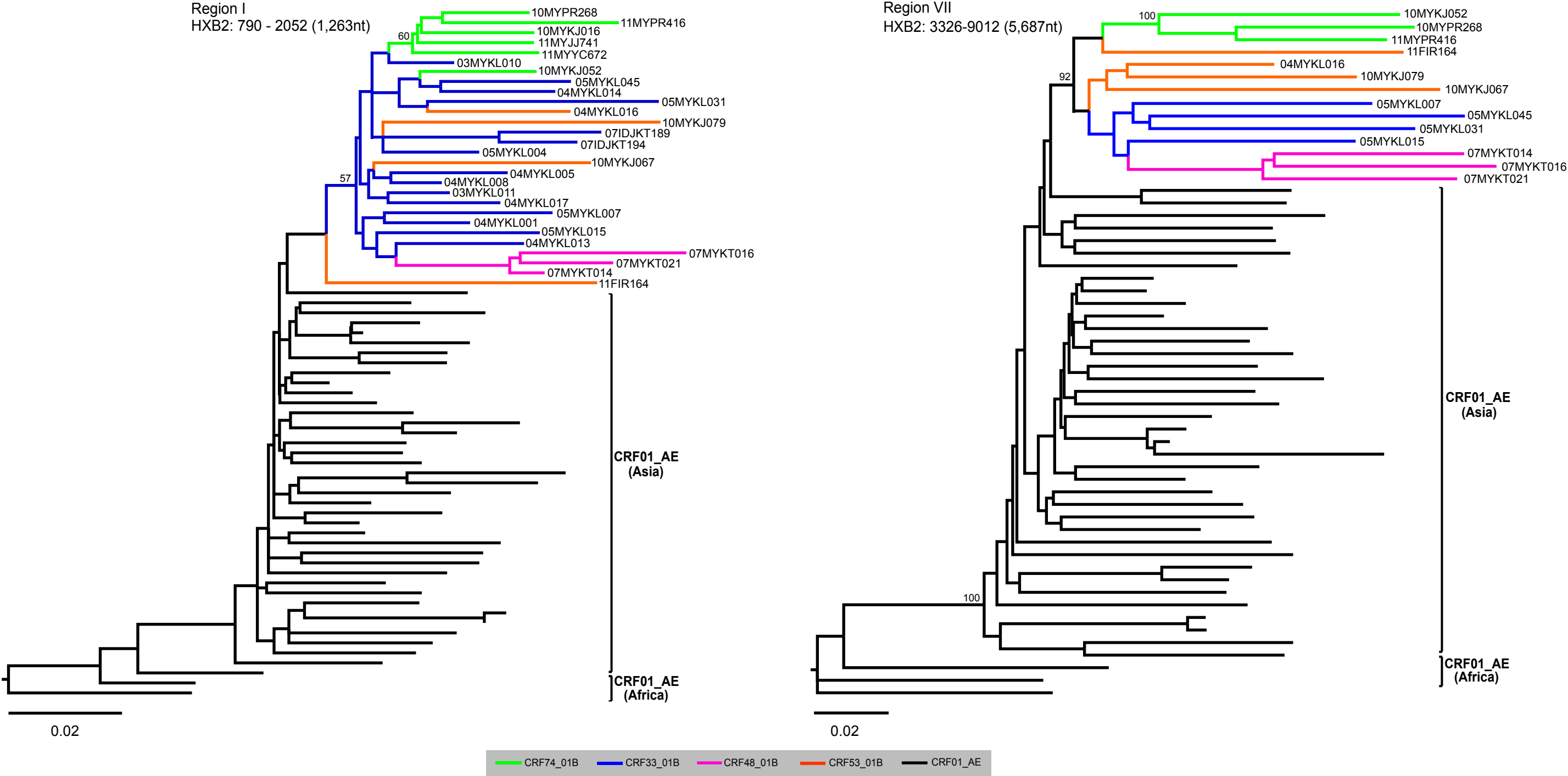

Supplement: S1 Fig — (PDF) [file pone.0133883.s001.pdf]
