# Supplementary material for: Genetic Characterization of a Novel HIV-1 Circulating Recombinant Form (CRF74_01B) Identified among Intravenous Drug Users in Malaysia: Recombination History and Phylogenetic Linkage with Previously Defined Recombinant Lineages
Source: PLoS One. 2015 Jul 21;10(7):e0133883. doi: 10.1371/journal.pone.0133883 (PMC4510129; doi:10.1371/journal.pone.0133883)
Supplement: S1 Table — (DOCX) [file pone.0133883.s002.docx]

**S1 Table. List of HIV-1 primers for near-full length genome amplification**

| **Region** | **Primer sequence (5' to 3')** | **HXB2 location** |
| --- | --- | --- |
| *gag* | ATCTCTAGCAGTGGCGCCCGAACAG  ACTCTTGCTTTATGGCTGGGTCC | 625 - 649  1874 - 1852 |
|  | CTCTCGACGCAGGACTCGGCTTGCT  CCTGACATGCTGTCATCATTTCTTCTA | 683 - 707  1843 - 1817 |
| *prot* | AAGGAACCCTTTAGAGACTATGTAGA  TATGGATTTTCAGGCCCAATTTTTG | 1657 - 1682  2716 - 2692 |
|  | GTAAAAAATTGGATGACAGAAACCTTG  ACTTTTGGGCCATCCATTCC | 1726 - 1752  2611 - 2592 |
| RT | GGAAACCAAAAATGATAGGGGGAATTGGAGG  CTGTACTTCTGCTACTAAGTCTTTTGATGGG | 2377 - 2407  3539 - 3509 |
|  | GTGGAAAAAAGGCTATAGGTACAG  CTGCCAACTCTAATTCTGCTTC | 2452 - 2475  3462 - 3441 |
| *pol* | TACACCAGACAAAAAGCATCAG  TCTACTTGTTCATTTCCTCC | 3194 - 3215  4193 - 4176 |
|  | GGCAACTTTGTAAACTCCTTAGGGGG  CCCTCCAAT TCCTTTGTGTGCTGGC | 3379 - 3404  4181 - 4157 |
| *int* | CTATGTAGATGGGGCAGCTA  TACTGCCCCTTCACCTTTCCA | 3869 - 3888  4976 - 4965 |
|  | AAGTAAACATAGTAACAGACTCAC  GCTGTCCCTGTAATAAACCCG | 4024 - 4047  4919 - 4899 |
| *vif* | TAAGACAGCAGTACAAATGGCAG  GGCTGACTTCCTGGATGGTTCCAGGGC | 4745 - 4767  5884 - 5858 |
|  | GGGGGGATTGGGGGGTACAGTGCAGGGG  AGGGCTCTAGGTTAGGATCTACCAGTTCCA | 4794 - 4821  5862 - 5833 |
| *vpu* | TTCATTTCAGAATTGGGTG  CTAGACTACCATTTAACAGCA | 5767 - 5786  7020 - 7000 |
|  | TTGGGTGTCAACATAGCAGAATAGG  AGTTGAGTTGATACCACTGGC | 5779 - 5803  7000 - 6980 |
| *env* | CTCCAGCTGGTTWTGCRATT  GTCTGGCCTGTACCGTCAGCG | 6880 - 6899  7851 - 7831 |
|  | CAGCTGGTTWTGCGATTCTAA  GCCCATAGTGCTTCCTGCTGCTCC | 6883 - 6903  7817 - 7794 |
| *gp41* | GACAAT TGGAGAAGTGAATT  AGACACTGCTCCTACTCCTTCTG | 7653 - 7672  8898 - 8876 |
|  | GGGCAAAGAGAAGAGTGGTGG  TTTGACCACTTGCCACCCAT | 7723 - 7743  8816 - 8797 |
| nef-3'LTR | GAGTTAGGCAGGGATACTTCAC  TAAGCACTCAAGGCAAGC | 8344 - 8364  9635 - 9618 |
|  | AGCCTGTGCCTCTTCAGCTACCA  GCACTCAAGGCAAGC TTTATTGAGGCT | 8508 - 8530  547 - 521/ 9632 - 9606 |
